# Residual Flexural Strength of Concrete Reinforced with Recycled Carbon Fibers from Wind Turbine Blades

**DOI:** 10.3390/ma18225195

**Published:** 2025-11-15

**Authors:** Julita Krassowska

**Affiliations:** Department of Building Structures, Bialystok University of Technology, 15-351 Bialystok, Poland; j.krassowska@pb.edu.pl

**Keywords:** sustainable concrete, fiber reinforcement, residual strength, circular economy, wind turbine blade recycling, carbon fibers

## Abstract

**Highlights:**

What are the main findings?

- Recycled carbon fibers enhance post-cracking strength of concrete.
- Fiber length and dosage strongly affect residual flexural behavior.
- New coefficients (*R_i_*, *E*) quantify fiber efficiency in concrete.

**What are the implications of the main finding?**

- 38–50 mm fibers at 8 kg/m^3^ ensure optimal crack control and ductility.
- Results support recycling wind turbine blades for structural concrete.
- Provides a path toward sustainable, high-performance fiber concretes.

**Abstract:**

The study aims to assess the potential of recycled carbon fibers recovered from end-of-life wind turbine blades as a sustainable reinforcement material for concrete and to establish correlations between fiber parameters and the mechanical behavior of fiber-reinforced concrete. The research focuses on how fiber length, content, and cement type affect the residual flexural strength and cracking behavior of FRC. The experimental program included 48 concrete mix series with varying fibre lengths (25, 38, and 50 mm), dosages (0, 2, 4, and 8 kg/m^3^), cement types (CEM I 42.5 and CEM II 42.5R/A-V), and water-to-cement ratios (0.50 and 0.40). Mechanical properties such as compressive strength, tensile strength, modulus of elasticity, and residual flexural strength were evaluated. Notched beams underwent three-point bending tests, and the progression of cracks was tracked using the digital image correlation method. The analysis revealed that enhancing both the fiber content and length generally bolstered the toughness and post-cracking characteristics of concrete, with a notable effect observed for fibers ranging from 38 to 50 mm in length when used at a dosage of 8 kg/m^3^. However, the effects depend on the fiber recovery technology and the base concrete strength, which may influence the results and should be considered as a limitation of this study.

## 1. Introduction

The global development of wind energy has led to a rapid increase in the number of installations, which over time reach the end of their technical service life [1]. One of the most problematic types of waste generated from turbine decommissioning are composite rotor blades, which can exceed 80 m in length. Due to their large volume, complex structure, and resistance to degradation, blades pose a serious challenge in terms of disposal and recycling. Currently, the dominant methods include landfilling, mechanical shredding, or co-incineration, but all of these are associated with significant environmental or technological costs. An alternative is the recovery of carbon or glass fibers from within the blade structure and their reuse in construction materials. Such an approach not only minimizes the amount of waste but also aligns with the concept of sustainable design, by enhancing the durability of concrete structures while simultaneously reducing the consumption of primary raw materials [2,3,4].

Processing methods of recycled carbon fibers can considerably improve the mechanical performance of concrete and cementitious composites. Nevertheless, their efficiency is strongly governed by the recovery parameters, including fiber length, cleanliness, degree of structural damage, and the presence of residual polymeric matrix [5].

**Figure 1:**
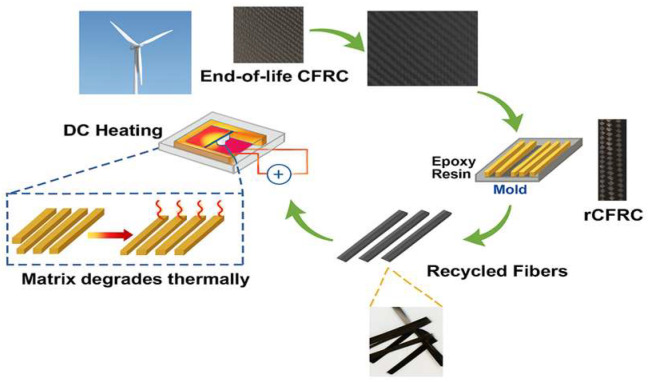
Scheme of carbon fiber recovery from wind turbine blades using resistive heating (DC Heating). Figure prepared by the Author, inspired by the concept presented [6].

**Figure 2:**
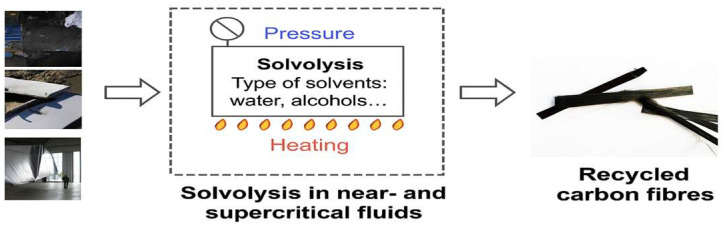
Chemical recycling process of composite waste. Figure modified by the Author based on [8].

**Figure 3:**
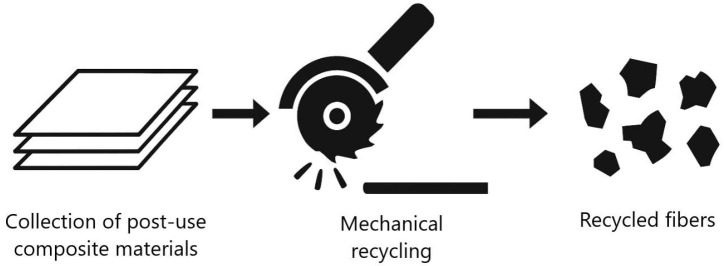
Mechanical recycling process of composite waste. Figure prepared by the Author, inspired by the concept presented [9].

Carbon fibers used in the structure of wind turbine blades are typically embedded in a polymer matrix (e.g., epoxy), which complicates their direct recovery and reuse. Consequently, several recycling technologies for carbon fiber-reinforced polymers (CFRPs) have been developed, with three main approaches commonly reported:

1. Thermal recycling (pyrolysis). This method involves the decomposition of the polymer matrix in a controlled atmosphere (most often nitrogen) at temperatures of about 500–600 °C. The process offers high efficiency, with recovery of up to 75% of the material mass. The fibers largely retain their geometry and suffer limited degradation, provided the parameters are properly optimized. However, potential drawbacks include partial surface damage, gas emissions, and high energy demand (Figure 1) [6].

2. Chemical recycling (solvolysis). This process involves depolymerization of the polymer matrix in a liquid medium using organic solvents or chemical agents, often under elevated pressure and temperature. The recovered fibers are of very high quality (clean, without resin residues) and exhibit high retention of their mechanical properties. However, the process requires costly equipment, is less easily scalable, and involves the use of potentially hazardous reagents (Figure 2) [7].

3. Mechanical recycling. This is the simplest and least expensive method, based on shredding (cutting, milling, grinding) of entire blades or their fragments. The resulting fibers exhibit significant variation in length and damage, contain residual matrix, and show limited quality and mechanical performance of the secondary material (Figure 3) [9].

Studies [10,11,12,13] have shown that the incorporation of recycled carbon fibers (recC) into concrete mixtures can significantly enhance flexural strength, ductility, and crack resistance, while simultaneously contributing to environmental goals by reducing the amount of composite waste sent to landfills. The literature [14,15] indicates that optimal dosage and appropriate surface modification of recycled carbon fibers (recC) can lead to an increase in flexural strength of up to 31–111% compared with plain concrete, while also improving durability and chemical resistance. Numerous studies [16] confirm that the addition of recC to concrete may enhance its flexural strength by 20% to as much as 111%, depending on fiber content, surface treatment, and mix composition. For instance, the incorporation of 0.5% recC resulted in a 31% increase in flexural strength, whereas surface-modified fibers achieved an improvement of up to 74.9%.

The primary mechanism of improvement is attributed to the crack-bridging effect of the fibers [13,17]. While flexural and tensile strengths are significantly enhanced, the effect on compressive strength is usually minor or slightly negative, with changes typically within ±5% [11,12]. On the other hand, ductility, impact resistance, and energy absorption capacity are consistently improved, resulting in a more continuous and ductile failure mechanism [12,15].

Nevertheless, research challenges remain concerning the uniform dispersion of fibers, their bond with the cementitious matrix, and their influence on compressive strength [11]. Concrete reinforced with carbon fibers reclaimed from end-of-life wind turbine blades into concrete represents not only a response to waste management issues but also a pathway towards the development of efficient and environmentally friendly construction materials.

Although the properties of concrete reinforced with steel, glass, or polypropylene fibers are well documented, the use of recycled carbon fibers in concrete structures remains relatively unexplored. Existing studies [11] have mainly focused on the technological aspects of fiber recycling and on basic mechanical properties such as compressive strength or overall durability. By contrast, only a limited number of works have addressed residual strength and the detailed fracture mechanisms of recC-reinforced concrete, particularly using advanced methods such as Digital Image Correlation (DIC). Comprehensive investigations are still missing on how fiber length and content, along with cement type and *w*/*c* ratio, govern crack formation and the post-cracking load-bearing response of concrete.

The aim of this study is to evaluate the behavior of concrete reinforced with recycled carbon fibers recovered from wind turbine blades. The study examined how fiber length, content, cement type, and the water-to-cement ratio influence post-cracking behavior, including load–CMOD response, residual flexural strength, and crack propagation characteristics. The research employed three-point bending tests [18] on notched beams combined with the Digital Image Correlation (DIC) technique, enabling precise observation of fracture development and strain distribution throughout successive loading stages. The findings offer valuable insights into the feasibility of using recycled carbon fibers for producing structural concretes. This study supports the development of sustainable construction materials and encourages the utilization of recycled resources within civil engineering applications.

## 2. Experimental Program

The research aimed to develop concrete mix formulations and to evaluate both the workability of fresh mixtures and the mechanical behavior of hardened concrete, including compressive, flexural, and residual flexural strength, as well as the modulus of elasticity. These evaluations were carried out to compare the performance of fiber-reinforced concretes with that of control mixtures.

Three groups of mixtures were prepared, each containing randomly distributed recycled carbon fiber strips of 25, 38, and 50 mm in length. Two cement types—CEM I 42.5 and CEM II 42.5R/A-V—were employed. The mixes were proportioned using two water-to-cement ratios (*w*/*c* = 0.50 and 0.40) and four fiber dosages (0, 2, 4, and 8 kg/m^3^, corresponding to 0, 0.11, 0.22, and 0.44% by volume), as presented in Table 1.

This experimental framework enabled a detailed investigation into the influence of fiber geometry, content, cement composition, and *w*/*c* ratio on the mechanical performance and durability of the tested concretes. In total, 48 mix series were produced, providing a comprehensive dataset for evaluating the effects of recycled carbon fiber reinforcement under varying technological conditions.

### 2.1. Materials

In this work, two base concrete mix compositions were developed, differing in water-to-cement ratios (*w*/*c* = 0.50 and 0.40) and cement type—CEM I 42.5R or CEM II 42.5R/A-V (320 kg/m^3^, Cemex Poland, Chełm, Poland). The detailed proportions of all components are listed in Table 2. In both mix series, recycled carbon fibers were incorporated at dosages of 0, 2, 4, and 8 kg/m^3^, partially replacing the fine aggregate by volume.

Recycled carbon fibers recovered from decommissioned wind turbine blades were used in this study. The fibers, introduced into the concrete as randomly dispersed bundles of 25, 38, and 50 mm in length, were produced via a two-stage thermal recycling process. The procedure consisted of pyrolysis conducted at 500 °C for 60 min in an inert atmosphere, followed by oxidation at 500 °C for 30 min in the presence of oxygen. This treatment efficiently eliminated the polymer matrix and yielded clean carbon filaments with retained structural integrity. After the recycling process, the material was mechanically cut using a guillotine system to the target lengths, with a cutting tolerance of approximately ±1 mm. The cutting accuracy was selectively verified using a caliper, confirming the correspondence between the obtained and intended fiber lengths. These fibers originated from decommissioned wind turbine blades composed of carbon-fiber-reinforced polymer composites used in large-scale energy structures. The recovered fibers demonstrated excellent mechanical performance, exhibiting an average tensile strength of 1168.5 MPa (within the range of 1093.5–1283.5 MPa) and an elastic modulus of 317 GPa, confirming their high stiffness. The mean ultimate strain at tensile failure was 0.395%, typical of brittle materials characterized by a high modulus of elasticity.

### 2.2. Testing Procedure

The workability of fresh concrete was evaluated using the slump test in accordance with EN 12350 [19], while the apparent density of hardened concrete was determined according to EN 12390-7 [20]. These measurements allowed assessment of the influence of fiber dosage, cement composition, and water-to-cement ratio on the workability and density of the mixes.

The compressive strength was evaluated following EN 12390-3 [21] on cube specimens (100 mm) and cylindrical specimens (150 × 300 mm) using a CONTROLS C86Z00 testing frame (Controls, Liscate, Italy) with a capacity of 3000 kN. The loading rate was maintained at 0.5 MPa/s. Flexural tensile strength was determined in line with EN 12390-5 [22] on 100 × 100 × 400 mm prisms under central point loading. The same testing system, with a maximum range of 100 kN, was used at a loading rate of 0.05 MPa/s. The static secant modulus of elasticity was obtained in accordance with EN 12390-13 [23] from 150 × 300 mm cylinders using the same 3000 kN press equipped with extensometers. Method B of loading was applied, and the modulus was calculated between 0.5 MPa and 40% of the ultimate compressive load.

**Figure 4:**
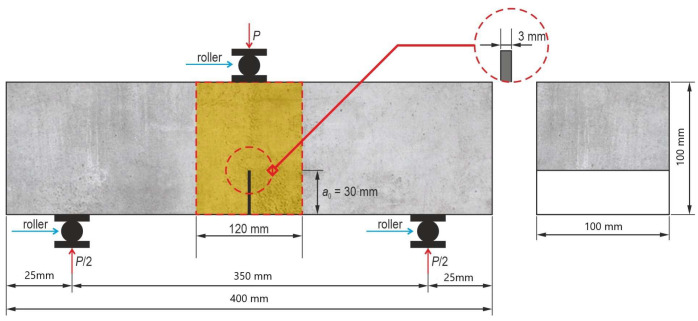
Geometry of the specimen for flexural tensile strength testing (mm).

Residual flexural tensile strength was tested according to modified procedure EN 14651 [18] according to Recommendation TC 50-FMT RILEM [24] on notched prisms measuring 100 × 100 × 400 mm (Figure 4). Although EN 14651 specifies monotonic loading, in this study a cyclic loading–unloading procedure was applied in accordance with the principles described in [24,25,26], to distinguish between the elastic (reversible) and inelastic (irreversible) components of CMOD deformation. The U-shaped notch was 30 mm deep, 5 mm wide, with a rounding radius of about 1.5 mm. The notches were cut one day prior to testing using a diamond saw. Each test series consisted of three specimens.

All samples were stored in water to prevent drying and shrinkage cracking. After 28 days, additional central notches (5 mm wide and 25 mm deep) were introduced across the entire prism width using a diamond saw.

The specimens were subjected to a central point load, while crack development was monitored using the Digital Image Correlation (DIC) technique and an extensometer measuring the crack mouth opening displacement (CMOD). The testing procedure consisted of loading up to 95% of the ultimate load, followed by four unloading–reloading cycles, and finally loading to complete failure of the specimen. The load was applied automatically, with CMOD serving as the key control parameter. Simultaneously with the CMOD measurements, the vertical displacement δ at the point of load application was recorded. The cyclic loading procedure was introduced to better characterize the post-cracking behavior of fiber-reinforced concrete and to observe the ability of fibers to transfer stresses and maintain load-bearing capacity after partial unloading. A single monotonic loading to failure does not allow for observing the gradual development of cracks and changes in the fiber–matrix interaction. The use of loading–unloading cycles enables a more controlled investigation of the phenomena occurring between the onset of cracking and the final failure of the specimen, providing deeper insight into the progressive damage and fiber bridging mechanisms [25]. The observed fluctuations in the load–CMOD diagrams correspond to these cyclic loading stages and reflect the progressive development and partial closure of cracks during each cycle.

The residual flexural tensile strength *f_Rj_* was determined according to modified EN 14651 [18] using the following equation:

(1) $$f_{R,j}=\frac{3P_{j}l}{2{bh}_{sp}^{2}}$$

where *P_j_*—load corresponding to CMOD = CMODj [N]; *l*—span of the beam; *b*—width of the beam; *h_sp_*—distance between the top of the notch and the top edge of the beam.

Cyclic loading/unloading was applied for research purposes to monitor crack evolution; residual flexural tensile strengths $f_{R1}$–$f_{R4}$ were calculated from the primary loading envelope at CMOD = 0.5, 1.5, 2.5, and 3.5 mm. This procedure is a non-standard modification of the normative test incorporating the principles of fracture mechanics described in Recommendation TC 50-FMT RILEM [24].

**Figure 5:**
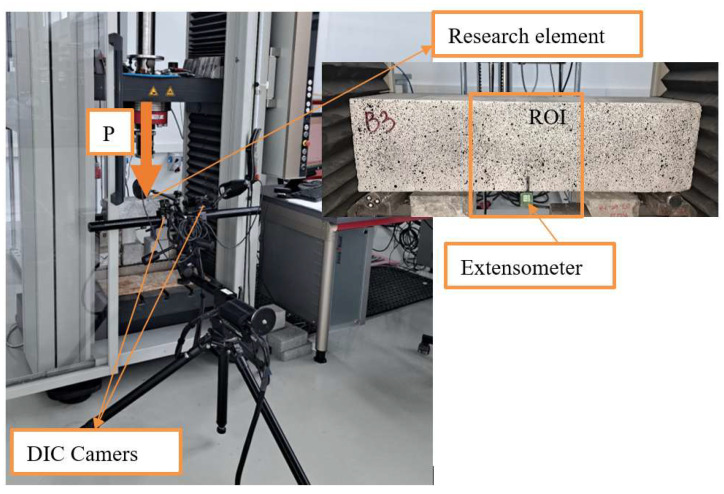
Schematic diagram of the DIC technique (DIC) test setup, showing the position of cameras, lighting, and the monitored region of interest (ROI) used for capturing surface deformations during the bending test.

To monitor the fracture process and assess the degree of damage, the Digital Image Correlation (DIC) technique was applied. The measurement system consisted of two synchronized cameras and a control unit. The cameras recorded images of the region of interest (ROI) at a frequency of 1 Hz, with a resolution of 5520 × 3680 pixels and a scale factor of 5.78 μm/pixel. The distance between the digital cameras and the monitored surface was 395 mm. For this purpose, the surface of the ROI was first coated with white paint as a background and then randomly sprayed with black acrylic paint to create a speckle pattern. Additional lighting was used to enhance the visibility of the speckle pattern. A schematic diagram of the test setup is shown in Figure 5.

## 3. Analysis and Results

This section summarizes the results of the tests performed on fresh and hardened concrete incorporating recycled carbon fibers. The evaluated parameters included compressive and tensile strength, Young’s modulus, and residual flexural strength.

The introduction of recycled carbon fibers led to a decrease in workability, shifting the slump class from S3 (100–150 mm) to S1 (10–40 mm) with increasing fiber dosage and length. This reduction is attributed to enhanced internal friction, fiber entanglement, and water absorption within the mix. The effects of cement type and water-to-cement ratio were relatively minor compared to those of fiber parameters. A small reduction in bulk density (approximately 2255–2360 kg/m^3^) was also noted at higher fiber contents, resulting from the lower intrinsic density of carbon fibers and slightly reduced mix compaction. More comprehensive experimental data and graphs are available in the previous study [5].

Table 3 summarizes the average strength values obtained for each concrete mix. The results provide a basis for identifying the influence of each variable on mechanical performance and enable comparison between fiber-reinforced and reference (non-reinforced) concretes. Each average value presented in Table 3 was determined based on a minimum of three test specimens for each concrete series.

### 3.1. Compressive Strength

The analysis of compressive strength results showed that all series exhibited high values of *f_c_* ranging from 49.1 to 59.4 MPa, corresponding to concrete class C40/50 and above. The addition of recycled carbon fibers (0.11–0.44% by volume) contributed to a gradual increase in strength—in most cases, a clear rising trend was observed with increasing fiber content. As an example, in the series utilizing 25 mm fibers and CEM I cement, the compressive strength rose from 51.60 MPa (as a reference) to 58.03 MPa at a concentration of 8 kg/m^3^, marking an increase of roughly 12%. A similar improvement was observed for 38 mm and 50 mm fibers, with the highest values recorded for mixtures with CEM II 42.5 R/B-M (S-V), reaching 59.09–59.37 MPa at 8 kg/m^3^.

The effect of fiber length was not very significant—both shorter fibers (25 mm) and longer ones (50 mm) improved fc, but the variations between the series were minimal, ranging from 1 to 2 MPa. Mixtures with a lower water-to-cement ratio (*w*/*c* = 0.40) showed slightly higher compressive strengths, confirming the positive effect of reduced mixing water.

Standard deviations were generally low (1–4 MPa), indicating good homogeneity and repeatability of the mixtures. Higher deviations (6–9 MPa) appeared only in isolated cases, likely due to minor inconsistencies in fiber dispersion at higher dosages. Overall, the results confirm that the type and amount of fibers had no significant influence on *f_c_*, which aligns with previous findings reported in the literature [27,28,29,30,31,32,33,34,35,36] (Table 4). Numerous studies have shown that different fiber types interact with the cement matrix primarily through surface adhesion and mechanical interlocking, leading to variations in the composite’s mechanical response.

The results obtained for carbon fibers from recycling (recC) demonstrate their high effectiveness in improving the compressive strength of concrete, particularly when optimally dosed and uniformly dispersed within the cementitious matrix. The largest increases, reaching up to 12% compared with the reference mixture, were achieved at dosages of around 8 kg/m^3^, corresponding to only 0.44% by volume. This efficiency, attained at relatively low fiber content, is comparable with values reported in the literature for basalt fibers and virgin carbon fibers, which at similar dosages produced strength gains of 10–11%. In comparison with steel fibers, which typically increase compressive strength in the range of 9–18% but require much higher dosages (1–3% by volume), recycled carbon fibers appear to be a more material-efficient solution. Polypropylene fibers, although beneficial in terms of rheological properties and ductility, are less effective in enhancing *f_c_*, usually providing 5–15% improvement at higher mass dosages.

### 3.2. Modulus of Elasticity

A study on the longitudinal secant modulus of elasticity for concrete (*E_c,sec_*) showed that all investigated mixtures reached values in the range of 39.09–51.78 GPa. However, the results were clearly affected by three main factors: cement type, fiber length, and fiber dosage.

The influence of recycled carbon fibers was nonlinear. In most cases, at low and medium dosages (2–4 kg/m^3^), a moderate increase in *E_c,sec_* of 3–5% was observed compared with the reference mixtures. At the peak dosage of 8 kg/m^3^, a decline in modulus was noted across several series. For instance, when using CEM I 42.5 with 25 mm fibers, the Ecm value rose from 47.82 GPa (reference) to 49.55 GPa at a fiber concentration of 4 kg/m^3^ but decreased to 44.79 GPa at 8 kg/m^3^. A similar pattern was noted for CEM II, indicating that higher fiber amounts might impede compaction, elevate porosity, and create local inconsistencies.

Cement type significantly influenced the modulus. Mixtures with CEM I 42.5 generally exhibited more stable and higher values, most often above 46 GPa. Concrete with CEM II 42.5 R/B-M (S-V) showed greater variability, ranging from 39.09 to 51.78 GPa. The presence of fly ash and other mineral additions in CEM II may have had a beneficial effect on the long-term structure of the matrix, while at high fiber dosages it could have reduced the initial stiffness of the material.

Fiber length also played a role. Shorter fibers (25 mm) produced more stable results, with smaller differences between dosages. Longer fibers (38 mm and 50 mm) led to greater fluctuations. For instance, in the mix with CEM I and 38 mm fibers, *E_c,sec_* dropped to 41.87 GPa at 4 kg/m^3^, indicating that longer fibers may have disrupted homogeneity and compaction of the mixture more strongly.

The standard deviations were usually low, in the range of 0.5–3.5 GPa, confirming good repeatability and reliability of the tests. Higher deviations (>4 GPa) were sporadic and occurred mainly in series with the highest fiber contents, likely due to reduced workability and non-uniform distribution.

The influence of fiber addition on the secant modulus of elasticity of concrete is a complex issue and remains inconsistently described in the literature. The present results are consistent with previous observations (Table 5). Depending on fiber type, dosage, length, and mix composition, both increases and decreases in Young’s modulus are possible. Steel fibers in moderate amounts may slightly improve stiffness [29,37], while synthetic fibers with a low modulus (such as PP or PVA) often reduce it, particularly at higher dosages [38,39,40]. Basalt fibers and hybrid micro–macro fiber [40,41,42] combinations, on the other hand, exhibit the greatest potential for stiffness enhancement when properly balanced in the mixture [36].

### 3.3. Flexural Strength

The analysis of flexural tensile strength results showed that the addition of recycled carbon fibers had a clear and beneficial effect on the performance of the tested concretes. In the reference mixtures (without fibers), *f_ctm_* values ranged from 2.04 to 3.87 MPa, while a proportional increase was observed with fiber content. At the peak dosage of 8 kg/m^3^, equivalent to 0.44% by volume, the flexural strength was observed to be between 5.12 and 7.87 MPa. The largest gain was recorded in the series with CEM II 42.5 R/B-M (S-V) and 25 mm fibers, where strength increased from 2.70 MPa to 7.87 MPa (an increase of ~191%), indicating very effective fiber anchorage within the cementitious matrix.

Fiber length proved to be an important factor. For 50 mm fibers, a strength increase was observed at maximum dosages (up to 6.97 MPa with CEM I and 6.54 MPa with CEM II), confirming the beneficial effect of longer bundles on load transfer after cracking. Shorter fibers (25 mm) provided a more uniform improvement across the entire dosage range, while medium fibers (38 mm) produced intermediate results.

Cement type also influenced performance. Concretes with CEM II 42.5 R/B-M (S-V) generally achieved higher *f_ctm_* values at higher fiber dosages, likely due to the presence of mineral additives improving fiber–matrix bond. By contrast, mixtures with CEM I 42.5 showed narrower result ranges and lower standard deviations.

Standard deviations were typically very low (0.01–0.89 MPa), indicating good repeatability. Higher deviations (>1 MPa) occurred only in a few series with high fiber contents, which may be attributed to local heterogeneity of fiber distribution in less workable mixes. Numerous studies [29,36,37,38,39,40,44,45,46,47] in the literature shown in Table 6 confirm that fibers significantly influence the flexural strength of concrete, with the scale of the effect depending primarily on fiber type, dosage, and orientation within the cementitious matrix.

Recycled carbon fibers (recC) represent a promising alternative to traditional types of dispersed reinforcement. At moderate dosages, they demonstrate effectiveness comparable to steel and basalt fibers, improving the load-bearing capacity and stiffness of concrete. Steel fibers are still considered the most effective—in UHPFRC, increases in flexural strength of up to 180% have been reported at 2% by volume [29,37]. Synthetic fibers (PP, PVA, polyolefin) are beneficial at low contents (0.15–0.2% by cement mass), enhancing flexural strength and fracture energy, although excessive amounts may reduce strength [36,46,47]. Basalt and glass fibers also improve flexural strength, while hybrid systems (e.g., basalt + PP, PVA + PP) can increase performance severalfold [38,39,40]. Against this background, recycled carbon fibers stand out as a material with high technical and environmental potential, combining some of the advantages of steel, basalt, and synthetic fibers, while addressing the critical aspect of sustainable waste utilization [44,45].

### 3.4. Splitting Strength

The evaluation of residual flexural tensile strength (*f_cl_*) revealed that the addition of carbon fibers from recycling markedly enhanced the post-cracking load-bearing capacity of concrete. The obtained values of *f_cl_* ranged from 2.30 to 6.87 MPa, exceeding the levels recorded for the reference specimens (2.30–3.27 MPa).

Even at low fiber dosages (2 kg/m^3^; ~0.11% by volume), a marked improvement was observed—on average 30–50% compared with the fiber-free mix. With increasing fiber content (4 and 8 kg/m^3^), this effect became more pronounced. For example, in the series with CEM II 42.5 R/B-M (S-V) and 38 mm fibers, *f_cl_* increased from 2.57 MPa to 6.87 MPa, corresponding to a gain of ~170%.

The highest *f_cl_* values were achieved with 38 mm and 50 mm fibers at the maximum dosage of 8 kg/m^3^. These fibers acted effectively as bridges across the tensile zone, restricting crack propagation and enhancing post-cracking capacity. Shorter fibers (25 mm) produced more uniform improvements by strengthening the matrix and limiting crack widths, although at higher dosages the effect was less pronounced.

Cement type also played an important role. Mixes with CEM I 42.5 showed stable, predictable increases in *f_cl_*, whereas concretes made with CEM II 42.5 R/B-M cement exhibited more dynamic gains, likely due to the presence of mineral additives enhancing fiber–matrix adhesion. A lower water-to-cement ratio (*w*/*c* = 0.40) further improved both the initial concrete strength and the efficiency of fibers after cracking, owing to a denser structure and improved stress transfer.

Standard deviations were generally very low (0.01–0.5 MPa), indicating high repeatability. Higher deviations (0.6–0.9 MPa) occurred only occasionally, mainly in series with high fiber contents where reduced workability could limit uniform fiber distribution.

Overall, the recycled carbon fibers demonstrated outstanding efficiency in enhancing splitting tensile strength. Increases of up to 170% were achieved at relatively low dosages (0.11–0.44% by volume), placing them on par with steel fibers, for which the literature reports [27,28] gains of up to 162% but at much higher dosages (0.5–2% by volume) (Table 7). Recycled carbon fibers also exhibited behavior similar to basalt and hybrid micro–macro fibers, providing effective crack bridging and post-cracking durability, while at lower dosages they resembled polyester or polypropylene fibers, though with typically higher efficiency [9,12,29,48]. Compared with other fiber types—polyester (+65.7% at 0.3% vol.) [40,41], polypropylene (+42.9% at 0.5% vol.) [40,41], basalt and hybrid (+22–29%) [12,42,49,50], or natural (~30–40% at 1.5% by mass) [36,51,52]—recycled carbon fibers deliver the highest reinforcement effect at the lowest dosage.

### 3.5. Load—Crack Mouth Opening Displacement (P-CMOD)

**Figure 6:**
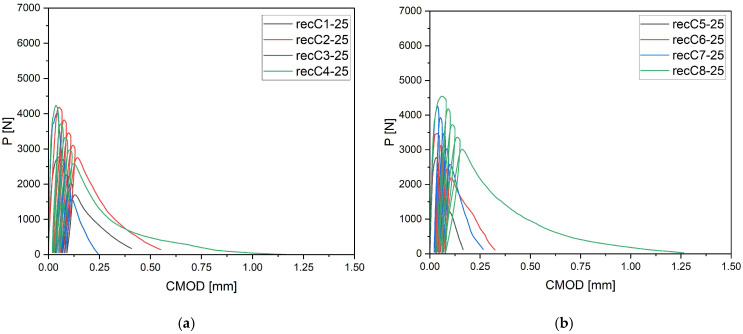

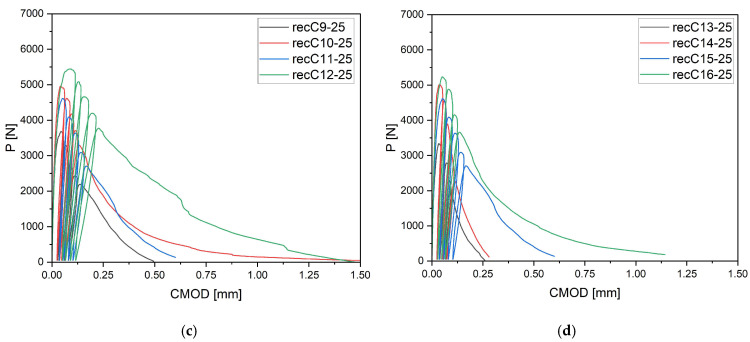
Load–CMOD curves for concrete specimens with recycled carbon fibers of 25 mm length (**a**) recC1-25 (0 kg/m^3^), recC2-25 (2 kg/m^3^), recC3-25 (4 kg/m^3^), recC4-25 (8 kg/m^3^) (**b**) recC5-25 (0 kg/m^3^), recC6-25 (2 kg/m^3^), recC7-25 (4 kg/m^3^), recC8-25 (8 kg/m^3^) (**c**) recC9-25 (0 kg/m^3^), recC10-25 (2 kg/m^3^), recC11-25 (4 kg/m^3^), recC12-25 (8 kg/m^3^) (**d**) recC13-25 (0 kg/m^3^), recC14-25 (2 kg/m^3^), recC15-25 (4 kg/m^3^), recC16-25 (8 kg/m^3^).

The P–CMOD curves shown in Figure 6 for concrete with 25 mm fibers indicate that short dispersed reinforcement improves the material’s behavior, but the effect is limited to the early stages of post-cracking. Compared to the reference specimens, where a sudden drop in load occurred immediately after crack initiation, the inclusion of 25 mm fibers allowed partial retention of load capacity and slowed down crack development. The difference was particularly evident at fiber contents of 4 and 8 kg/m^3^, where bridging was more pronounced and the load drop was less abrupt. Nevertheless, as CMOD increased, the curves gradually declined and residual forces reached low values. This suggests that shorter fibers act primarily as a mechanism delaying sudden loss of capacity, but do not provide long-term stabilization at larger deformations. They can therefore be considered effective in enhancing concrete ductility at the stage of crack initiation, but less efficient in controlling crack propagation.

**Figure 7:**
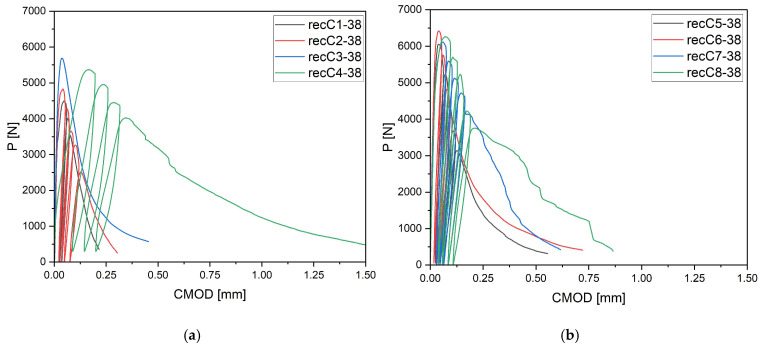

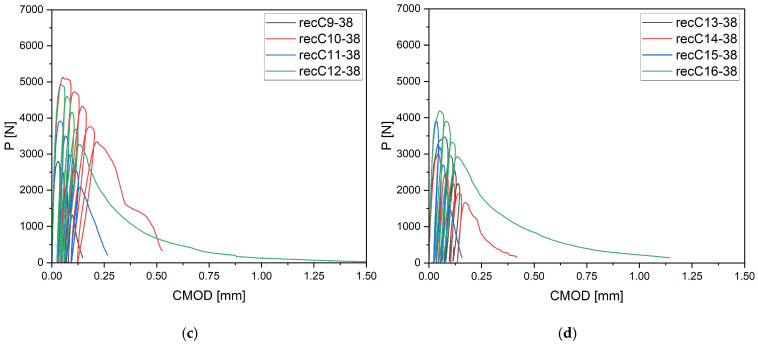
Load–CMOD curves for concrete specimens with recycled carbon fibers of 38 mm length (**a**) recC1-38 (0 kg/m^3^), recC2-38 (2 kg/m^3^), recC3-38 (4 kg/m^3^), recC4-38 (8 kg/m^3^) (**b**) recC5-38 (0 kg/m^3^), recC6-38 (2 kg/m^3^), recC7-38 (4 kg/m^3^), recC8-38 (8 kg/m^3^) (**c**) recC9-38 (0 kg/m^3^), recC10-38 (2 kg/m^3^), recC11-38 (4 kg/m^3^), recC12-38 (8 kg/m^3^) (**d**) recC13-38 (0 kg/m^3^), recC14-38 (2 kg/m^3^), recC15-38 (4 kg/m^3^), recC16-38 (8 kg/m^3^).

The behavior of concrete with 38 mm carbon fibers is presented in Figure 7. Medium-length fibers exhibited a more balanced influence—both at the moment of crack initiation and during the subsequent crack development phase. Even at low dosages, the fibers extended the period during which the concrete remained stable after crack initiation. At higher dosages (4–8 kg/m^3^), this effect became dominant: the post-cracking curves did not show abrupt drops in load but maintained relatively high residual load levels over a longer CMOD range. This indicates more effective crack bridging and better interaction with the cement matrix compared to shorter fibers. Importantly, 38 mm fibers provided a compromise by ensuring stable post-cracking behavior while not reducing workability and homogeneity of the mix to the same extent as longer fibers. From an engineering perspective, they can therefore be regarded as the optimal fiber length, combining mechanical efficiency with acceptable technological performance.

**Figure 8:**
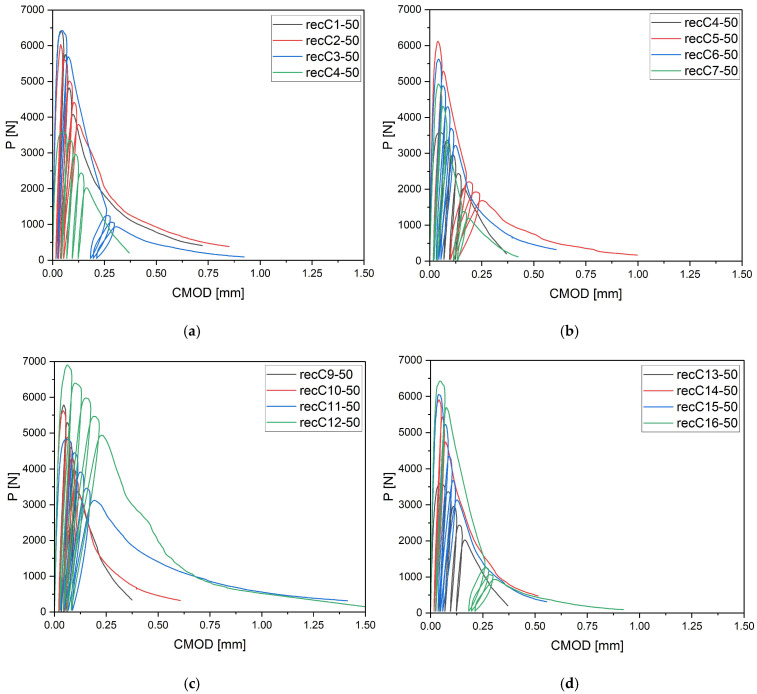
Load–CMOD curves for concrete specimens with recycled carbon fibers of 50 mm length (**a**) recC1-50 (0 kg/m^3^), recC2-50 (2 kg/m^3^), recC3-50 (4 kg/m^3^), recC4-50 (8 kg/m^3^) (**b**) recC5-50 (0 kg/m^3^), recC6-50 (2 kg/m^3^), recC7-50 (4 kg/m^3^), recC8-50 (8 kg/m^3^) (**c**) recC9-50 (0 kg/m^3^), recC10-50 (2 kg/m^3^), recC11-50 (4 kg/m^3^), recC12-50 (8 kg/m^3^) (**d**) recC13-50 (0 kg/m^3^), recC14-50 (2 kg/m^3^), recC15-50 (4 kg/m^3^), recC16-50 (8 kg/m^3^).

Figure 8 provides the analysis results for concrete with 50 mm carbon fiber reinforcement. The longest fibers clearly stand out in terms of their ability to stabilize the cracking process. The P–CMOD curves show that at higher dosages (4–8 kg/m^3^), load-bearing capacity was maintained at very high levels even at large crack openings. Concrete with 50 mm fibers not only exhibited a slower decline in load but also demonstrated the ability to sustain residual loads over a prolonged period—considerably more effectively than with shorter fibers. This behavior results from the formation of stronger bonds between the fibers and the cement matrix, which enables more efficient crack bridging and delays crack propagation. At the same time, it should be noted that fibers of this length increase the risk of technological issues: the mix becomes less homogeneous, and the risks of segregation and reduced workability rise. Therefore, 50 mm fibers should be regarded as a highly effective mechanical solution, but one that requires careful control of the mixing process. From the perspective of post-cracking behavior, 50 mm fibers represent the most effective variant among the studied lengths. The observed differences in the width and magnitude of the loading–unloading cycles (Figure 8a–d) result from the variable stiffness and post-cracking behavior of concretes containing different types and dosages of fibers. The same testing protocol was applied in all cases—four loading–unloading cycles up to 95% of the maximum load, followed by a fifth cycle leading to specimen failure. The variation in cycle length reflects the material’s ductility and fiber-bridging efficiency rather than differences in the testing procedure.

In light of the available literature [3,9,12,30,42,53,54,55,56,57] shown in Table 8, these results are positioned at a comparable or even higher level than for other fiber types, such as:

- Synthetic fibers (e.g., PP, PVA)—also increase residual strength, although their efficiency is often limited at higher dosages due to reduced workability. For instance, PP at 2.5% by volume increased flexural strength by 26.9%, with a toughness index of 4.75 [54,58].
- Basalt fibers—provide a flexural strength increase in the range of 20.8–43.5% at optimal dosages of 0.1–0.5% by volume. They are characterized by high thermal resistance and toughness indices of up to 4.64 [9,30,42,53].
- Glass fibers—deliver a comparable level of reinforcement to basalt fibers, with typical increases in *f_cf_* of up to 27.9% and toughness indices up to 4.86, while offering better dispersion uniformity [55,56].
- Virgin carbon fibers—according to [59], outperform all other fiber types in terms of residual and flexural strength, particularly in high-strength concretes [3,12,57,59,60].

**Figure 9:**
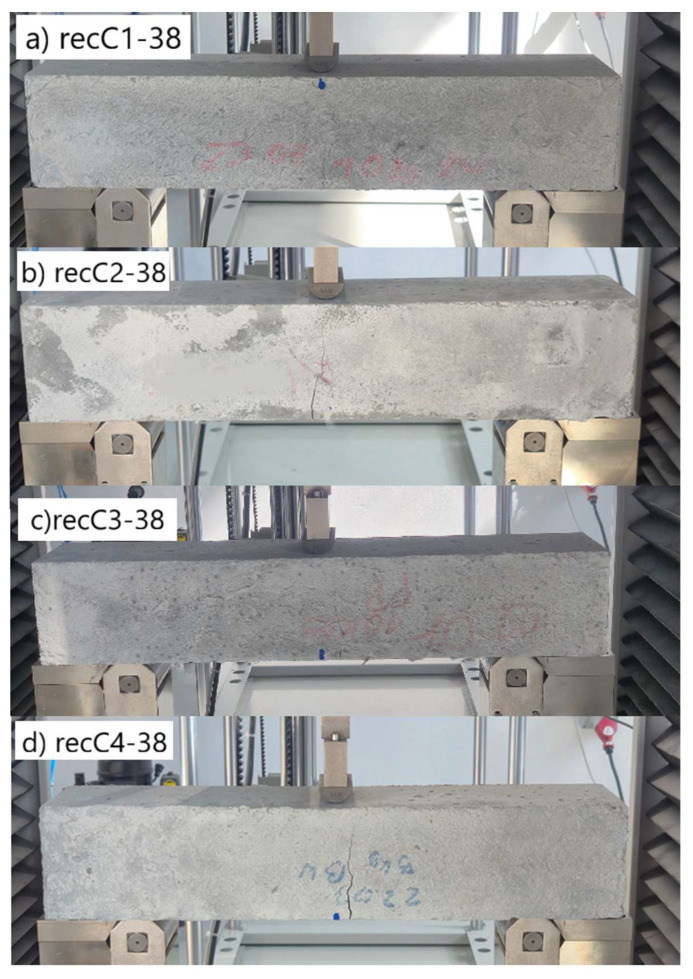
Crack pattern of concrete beams in series: (**a**) recC1-38 (0 kg/m^3^), (**b**) recC2-38 (2 kg/m^3^), (**c**) recC3-38 (4 kg/m^3^), (**d**) recC4-38 (8 kg/m^3^).

When comparing these findings with the results of the present study in Table 8, it can be concluded that recycled carbon fibers, despite being of lower quality than virgin fibers, exhibit reinforcing efficiency equal to or higher than synthetic, basalt, and glass fibers—both in terms of the increase in strength and in the improved post-cracking behavior of concrete. Figure 9 shows the crack development of beams reinforced with 38 mm fibers at dosages ranging from 0 to 8 kg/m^3^ under four-point bending. In the reference specimens (recC1-38) without fibers, a typical brittle P–CMOD response was observed, characterized by a sudden loss of load-bearing capacity after reaching the peak force. The inclusion of fibers resulted in a clear delay in crack development. The crack-bridging effect intensified with increasing fiber content, as confirmed by smoother curve profiles and higher load levels at larger CMOD values.

### 3.6. Residual Flexural Strength

**Figure 10:**
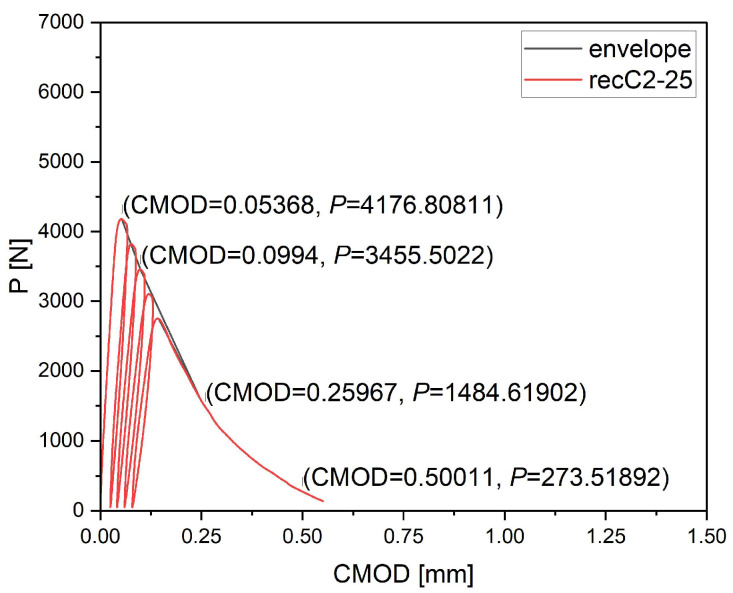
Example of the load *P*–CMOD curve for specimen recC2-25 with cyclic loading and unloading and envelope.

The results presented in the load–CMOD curves (Figure 6, Figure 7 and Figure 8) and summarized in Section 3.5 demonstrate consistent quantitative trends across all tested series. During cyclic loading, each specimen exhibited several loading–unloading loops on the load–CMOD curve. The envelope of the primary loading branches was constructed by connecting the maximum load points from each loading phase (Figure 10). This envelope represents the effective material response under increasing CMOD and was used to determine the residual flexural tensile strengths $f_{R1}$–$f_{R4}$ in accordance with the principles of EN 14651 [18].

**Figure 11:**
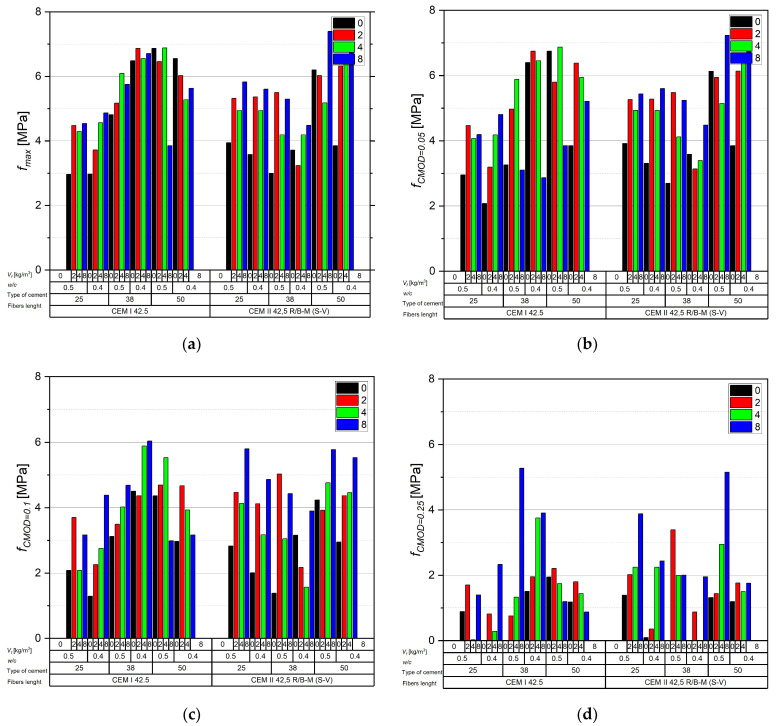

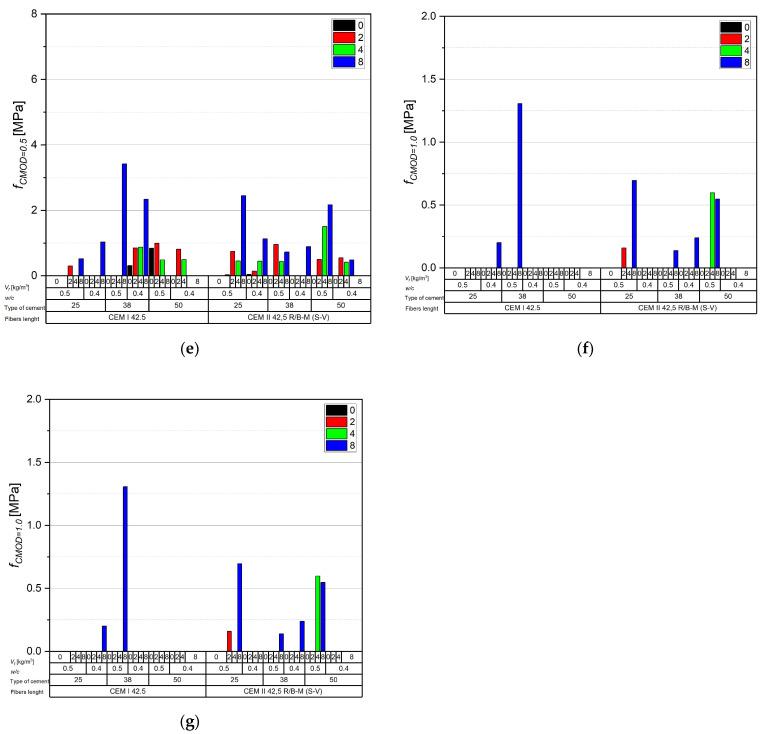
The relationship between the load (*P*) and the crack mouth opening displacement (CMOD) for concretes modified recycled carbon fibers in (**a**) *P* = max, (**b**) CMOD = 0.05 mm, (**c**) CMOD = 0.1 mm, (**d**) CMOD = 0.25 mm, (**e**) CMOD = 0.5 mm, (**f**) CMOD = 1.0 mm, (**g**) CMOD = 1.5 mm.

The maximum load $f_{max}$ falls within 2.97–7.40 MPa and increases clearly as the fiber dosage rises from 0 to 4 kg/m^3^; at 8 kg/m^3^ a plateau or a local decrease is often observed, attributable to reduced workability and poorer dispersion (Figure 11). In comparative terms, longer fibers (38–50 mm) consistently outperform 25 mm, which is particularly evident at larger crack openings, when pull-out and interfacial friction dominate over simple anchorage. Lowering the water–cement ratio from 0.50 to 0.40 produces a systematic gain in both $f_{max}$ and $f_{R,j}$ of ~0.3–0.8 MPa, reflecting a denser matrix and improved fiber–matrix bond. For comparable mixtures, CEM I 42.5 typically yields values higher by ~0.2–1.0 MPa than CEM II 42.5 R/B-M (S-V), indicating the influence of hydration kinetics and fines gradation on bridging efficiency.

For residual capacity, a predictable step-down is observed with increasing CMOD. At CMOD = 0.05 mm, values rise from reference ~2.1–3.9 MPa to ~5–7 MPa for 38–50 mm and ≥4 kg/m^3^ (with several configurations exceeding 6.5 MPa), effectively limiting the force drop beyond proportionality. At CMOD = 0.10 mm, typical levels are ~3–6 MPa for 38–50 mm and ≥4 kg/m^3^, preserving functional crack bridging. At CMOD = 0.25 mm, the range decreases to ~1–4 MPa; 25 mm fibers become largely ineffective, while 38–50 mm at 4–8 kg/m^3^ retain measurable capacity. At CMOD = 0.50 mm, significant values are sustained mainly with longer fibers and higher dosages (local maxima around ~5.2 MPa, though most mixes remain below ~2 MPa). At CMOD = 1.0–1.5 mm, residual strengths are generally low (usually ≤1.5 MPa, sporadically up to ~3.4 MPa), confirming the dominance of pull-out/rupture over bond and underscoring that serviceability is governed by small to medium crack openings (≤0.5 mm). Differences between cements persist at larger CMOD: CEM I favors more stable $f_{R,j}$ at intermediate openings, whereas CEM II shows greater sensitivity to dosage and fiber length.

From a design and production standpoint, the most effective compromise is 38–50 mm fibers at ≈4 kg/m^3^ with *w*/*c* = 0.40—a configuration that provides high $f_{max}$ and useful $f_{R,j}$ for CMOD ≤ 0.5 mm. Increasing the dosage to 8 kg/m^3^ may yield additional reserve at small–medium CMOD, but requires rigorous control of rheology and dispersion procedures to avoid clumping and segregation. For elements approaching ultimate states, values at CMOD ≥ 1.0 mm should be treated as limited and auxiliary.

### 3.7. Residual Reinforcement Coefficient (Ri)

To evaluate the effectiveness of fiber reinforcement at different stages of crack development, the residual reinforcement coefficient $R_{i}$ was proposed in this study by the Author. It is defined as the ratio of the residual strength at a given crack mouth opening displacement (CMOD = *i*) to the residual strength at the very early stage of cracking (CMOD = 0.05 mm), according to the equation:

(2) $$R_{i}=\frac{f_{CMOD=i}}{f_{CMOD=0.05}}$$

where $f_{CMOD=i}$—residual strength at CMOD = *i*; $f_{CMOD=0.05}$—residual strength at CMOD = 0.05 mm.

**Figure 12:**
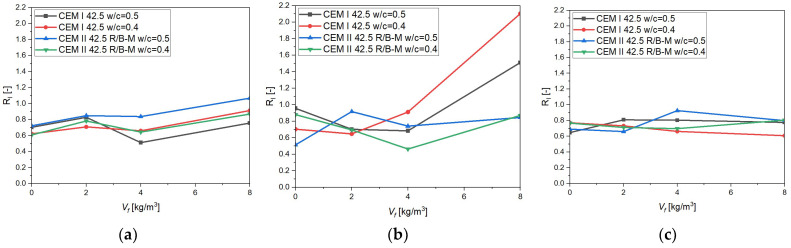
Residual reinforcement coefficient *R_i_* (0.1 mm) depending on fiber content (*V_f_*) for different cement types, fiber lengths, and water-to-cement ratios: (**a**) 25 mm fibers, (**b**) 38 mm fibers, (**c**) 50 mm fibers. (Lines are used only to illustrate general trends and do not represent continuous or interpolated data between independent concrete series).

This coefficient enables a quantitative analysis of load-bearing capacity loss as a function of crack propagation and allows assessment of the durability and effectiveness of fiber reinforcement in later stages of structural behavior. The analysis was carried out for CMOD = 0.1 mm and is presented in Figure 12. The calculated $R_{i}$ makes it possible to compare different fiber lengths, dosages, cement types, and water-to-cement ratios, thus providing a more comprehensive view of the efficiency of fiber reinforcement under crack development. This approach is consistent with methods reported in the literature [61,62].

The analysis of the residual reinforcement coefficient *R_i_* (0.1 mm) depending on fiber content (Figure 12a–c) revealed an influence of fiber length on the post-cracking behavior of concrete. Short fibers (25 mm) improved crack initiation resistance and enhanced residual load-bearing capacity even at low dosages (2–4 kg/m^3^), but their effectiveness was limited at larger crack openings. Medium-length fibers (38 mm) proved to be the most effective—particularly in combination with CEM I at a low *w*/*c* = 0.4, where *R_i_* values above 2.0 were obtained at 8 kg/m^3^, demonstrating a very strong bridging effect and a high ability to sustain post-cracking capacity. The longest fibers (50 mm) acted primarily as stabilizers—*R_i_* values remained within a narrow range of 0.7–0.85, without sharp increases, but with a clear effect of mitigating load capacity loss. These results indicate that 38 mm fibers provide the best compromise between mechanical efficiency and mix stability, while 25 mm and 50 mm fibers serve primarily as initial protection against sudden cracking and as stabilizers under large deformations, respectively.

### 3.8. Fiber Efficiency Coefficient

To quantitatively assess and compare the effectiveness of recycled carbon fibers in improving the post-cracking behavior of concrete, the Fiber Efficiency Coefficient (*E*) was proposed by the Author.

The coefficient is defined as the increment of residual load-bearing capacity per unit of fiber content, measured at a significant crack opening. It was determined according to the following equation

(3) $$E\;=\frac{\left(R_{1/0.25}-R_{1/0.05}\right)}{V_{f}}$$

where $R_{1/0.25}$—residual load measured at a crack opening CMOD = 0.25 mm; $R_{1/0.05}$—residual load measured at a crack opening CMOD = 0.05 mm; $V_{f}$—fiber content.

**Figure 13:**
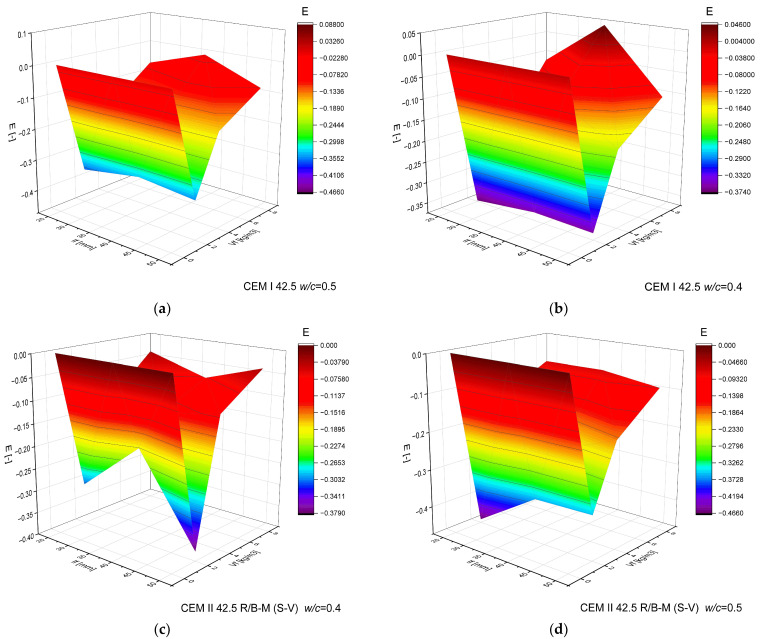
Fiber Efficiency Coefficient (E) depending on fiber length and fiber content (*V_f_*) for concretes with: (**a**) CEM I 42.5, *w*/*c* = 0.5; (**b**) CEM I 42.5, *w*/*c* = 0.4; (**c**) CEM II 42.5 R/B-M (S-V), *w*/*c* = 0.5; (**d**) CEM II 42.5 R/B-M (S-V), *w*/*c* = 0.4. (The surface plots represent a rough and illustrative approximation of discrete experimental data, intended to visualize general trends rather than precise interpolations).

The coefficient *E* describes the relative increase in residual capacity attributable to fibers (depending on their length, dosage, and effect compared with the reference concrete), according to Equation (3). The results are presented in Figure 13.

The highest values of the fiber efficiency coefficient were recorded for concrete made with CEM I 42.5 cement, using 38 mm fibers at *w*/*c* = 0.5 and a dosage of 8 kg/m^3^, reaching +0.088. At a lower 0,4 water to cement ratio, the same fiber length and dosage also produced a positive value (+0.045), confirming the favorable effect under optimized mix parameters. For 25 mm fibers, no positive effect was observed, indicating that medium fiber length is a key factor influencing efficiency. A clear advantage was noted for mixtures with CEM I over those with CEM II/B-M (S-V), which may suggest better compatibility of carbon fibers with a cement matrix containing fewer pozzolanic additives. Reducing the water-to-cement ratio from 0.5 to 0.4 did not significantly increase efficiency but contributed to more stable results, with lower variability of coefficient values.

### 3.9. Analysis of DIC

To comprehensively evaluate the behavior of concrete reinforced with recycled carbon fibers, an analysis of crack propagation was performed using the Digital Image Correlation (DIC) technique. Principal strain maps obtained from this system enabled a visual assessment of crack development depending on cement type, fiber dosage, fiber length, and water-to-cement ratio (*w*/*c*). The representative strain distributions at different loading levels (0.1 *P_max_*, 0.5 *P_max_*, 0.9 *P_max_*, and *P_max_*) are summarized in Table 9, which presents the principal strain maps on the concrete surface, showing the effect of fiber parameters and mixture composition on the development of surface cracking.

As expected, the reference specimens (without fibers), e.g., recC1-25, recC5-25, recC9-25, and recC13-25, exhibited very rapid and straight crack development of brittle character, localized along the notch axis. Already at 0.5 *P_max_*, cracks penetrated the entire cross-section, and at *P_max_* complete opening and failure of the element occurred. The introduction of dispersed reinforcement in the form of recycled carbon fibers resulted in a noticeable modification of the fracture mechanism. In fiber-reinforced series (e.g., recC4-25, recC8-25, recC12-25, recC16-25), delayed crack initiation, irregular crack paths, bridging effects—zones where fibers restricted crack opening—and strain dissipation were observed. Instead of one dominant crack, areas of distributed deformation developed.

Fiber length significantly influenced the efficiency of crack-propagation control. Fibers of 25 mm (e.g., series recC1–recC4) acted mainly at the initial stage of crack growth. Although bridging effects occurred (e.g., recC3-25, recC4-25), at *P_max_* strains were concentrated and the crack developed along one dominant path. Fibers of 38 mm (e.g., recC3-38, recC4-38, recC8-38) showed better performance—cracks were zigzag-shaped and dispersed, and deformations spread over a larger area. In series recC8-38, the most extensive strain distribution was noted, which preserved specimen integrity up to *P_max_*. Fibers of 50 mm (e.g., recC4-50, recC8-50) provided very high resistance to crack propagation. Already at 0.5 *P_max_*, strains were minimal (e.g., recC8-50), and even at *P_max_* crack development was effectively restrained. Reducing the *w*/*c* ratio from 0.5 to 0.4 increased resistance to crack propagation due to a denser matrix microstructure. Comparison of series recC4-25 and recC8-25 (*w*/*c* = 0.5) with recC4-25 and recC8-25 (*w*/*c* = 0.4) showed lower intensity of local strains, a more dispersed crack pattern, and an extended phase of performance without full crack opening. Similar effects were observed for 38 mm and 50 mm fibers—especially in recC7-38 and recC8-38, where at *w*/*c* = 0.4 cracks developed later and slower, and specimens retained residual capacity even under high loads.

The use of CEM II 42.5 R/B-M (S-V) resulted in a more stable but slightly less intense bridging effect compared to CEM I. For example, series recC12-25, recC16-25, recC12-38, and recC16-50 exhibited uniform strain distributions and less abrupt crack openings, although maximum deformation values were often lower than in corresponding CEM I series. This indicates favorable fiber–matrix interaction, albeit with somewhat lower efficiency under very large deformations. Observations at peak load showed significant differences in fracture behavior. Fiber-free specimens (e.g., recC1-25, recC5-25, recC13-38) failed suddenly in a quasi-brittle manner, with a single dominant crack. Specimens with 2–4 kg/m^3^ fibers (e.g., recC3-25, recC3-38, recC3-50) exhibited visible bridging effects, though crack openings remained significant. Specimens with 8 kg/m^3^ fibers (e.g., recC4-38, recC8-50, recC16-50) displayed the most desirable behavior: zigzag, irregular, and dispersed cracks, uniform strain distribution, and absence of abrupt failure.

## 4. Conclusions

This study examined the mechanical and fracture behavior of concrete reinforced with recycled carbon fibers obtained from end-of-life wind turbine blades. The experimental program investigated the effects of fiber length, dosage, cement type, and water-to-cement ratio on compressive, tensile, and flexural properties, as well as on crack propagation mechanisms. The main conclusions are as follows:

- Fiber-reinforced concretes achieved high compressive strength (49.1–59.4 MPa). Strength increased slightly with higher fiber dosage, and the best performance was obtained for 50 mm fibers with CEM II 42.5 R/B-M (S-V) cement at 8 kg/m^3^. The influence of fiber length on compressive strength was minor (1–2 MPa difference). The amount of fibers did not have a significant effect on compressive strength.
- The secant modulus of elasticity (*E_c,sec_*) showed moderate variation with fiber addition. At lower dosages (2–4 kg/m^3^), a slight increase in stiffness was observed, while at higher dosages (8 kg/m^3^) a small decrease occurred, likely due to the reduced homogeneity of the mix. This indicates the existence of an optimal fiber dosage range that improves effective stress transfer.
- The splitting tensile and flexural strengths increased significantly with higher fiber content, confirming the reinforcing and crack-bridging function of the fibers. Strength gains of up to 170% were achieved compared with reference concrete, particularly for 38–50 mm fibers at 8 kg/m^3^. It can be concluded that fiber length is a key parameter determining the fibers’ effectiveness in enhancing the concrete’s ability to carry tensile stresses.
- Load–CMOD curves showed that the presence of fibers delayed crack initiation and reduced the force drop beyond the proportional limit, leading to a more ductile post-cracking response. The failure of fiber-free beams was brittle and abrupt, in contrast to fiber-reinforced specimens, which were able to carry load even with increasing crack width (CMOD) and exhibited noticeable ductility. The most favorable behavior was obtained for 38 mm fibers at 8 kg/m^3^, providing an optimal balance between workability and crack-bridging efficiency.
- The analysis of residual flexural strength confirmed that fiber reinforcement was most effective at small and medium crack openings (CMOD ≤ 0.5 mm), demonstrating the ability of recycled carbon fibers to transfer tensile stresses after matrix cracking. To quantify this effect, the residual reinforcement coefficient (*R_i_*) was introduced, defined as the ratio between the residual flexural strength at a given crack opening (CMOD = *i*) and that was recorded at CMOD = 0.05 mm. For 38 mm fibers at 8 kg/m^3^, *R_i_* exceeded 2.0, indicating a strong crack-bridging effect and effective stress transfer during crack propagation. This parameter provides a clear, quantitative criterion for comparing different fiber geometries and mix compositions in terms of post-cracking behavior.
- The Fiber Efficiency Coefficient (*E*) was introduced to more accurately evaluate the effectiveness of recycled carbon fibers in enhancing the post-cracking behavior of concrete. The coefficient was defined as the increase in residual load-bearing capacity per unit of fiber content, determined from the difference between residual strengths at CMOD = 0.25 mm and CMOD = 0.05 mm. The analysis showed that *E* strongly depends on fiber length and cement type. The highest efficiency was obtained for 38 mm fibers at 8 kg/m^3^ in concretes with CEM I 42.5 and *w*/*c* = 0.5, where *E* reached +0.088, indicating a quantifiable improvement in post-cracking capacity. Short fibers (25 mm) exhibited negligible efficiency, while medium-length fibers provided the best balance between dispersion, interfacial bonding, and stress transfer. The results confirm that the coefficient *E* can serve as a quantitative criterion for comparing different fiber lengths, dosages, and cement matrices in terms of their contribution to the residual load-bearing capacity of fiber-reinforced concrete.
- Principal strain maps obtained from the DIC method showed that fiber reinforcement promoted more distributed and irregular crack propagation patterns.

The research objectives were achieved, confirming the feasibility of using recycled carbon fibers as an efficient and sustainable reinforcement for concrete. The results demonstrate that fiber length is a critical design parameter, as longer fibers (38–50 mm) provide superior crack-bridging efficiency. This finding may guide the optimization of industrial fiber dimensions for structural and precast concrete applications requiring enhanced ductility and crack control.

However, two important limitations must be acknowledged: the relatively high compressive strength of the reference concrete (≈C50) may have partially masked the reinforcing effect of fibers, and no direct comparison with commercially available virgin fibers was performed. Future research will address these aspects by including parallel tests on conventional fibers and extending the investigation to large-scale structural elements and durability performance.

## Figures and Tables

**Table 1:** Test Series.

Cement	CEM I 42.5		CEM II 42.5R/A-V	
Water to cement ratio	*w/c* = 0.5		*w/c* = 0.4	
*V_f_* [kg/m^3^]	0	2	4	8
Recycled carbon fibers recC	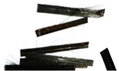		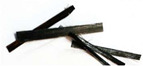	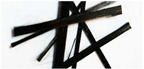
*l_f_* [mm]	***25***		***38***	***50***
*d_f_* [mm]	0.8		0.8	0.8

**Table 2:** Mixture proportions for the concrete.

Mixture Proportions	*w/c* = 0.5	*w/c* = 0.4
CEM I or CEM II, [kg/m^3^]	320	320
Water, [kg/m^3^]	160	128
Sand 0.125–2 mm, [kg/m^3^]	732	742
Aggregate 2/16, [kg/m^3^]	1203	1203
Sika Sikacem Superplast [kg/m^3^]	3.2	6.4

**Table 3:** Measured concrete parameters (compressive strength *fc*, splitting tensile strength *fc_l_,* tensile strength in bending *fc_tm_*, and longitudinal secant modulus of elasticity of concrete *E_c,sec_*).

Series	Type of Cement	*w/c*	*l_f_*	*V_f_*	*V_f_*	*fc*		*f_ctm_*		*f_cl_*		*E_c,sec_*	
	[-]	[-]	[mm]	[kg/m^3^]	[%]	[MPa]		[MPa]		[MPa]		[MPa]	
recC1-25	CEM I 42.5	0.5	25	0	0	51.60	±6.32	2.63	±0.07	2.30	±0.57	47.82	±1.20
recC2-25		0.5		2	0.11	52.10	±2.10	3.71	±0.12	3.31	±0.14	46.52	±0.78
recC3-25		0.5		4	0.22	57.47	±4.87	4.21	±0.60	4.25	±0.78	48.16	±1.86
recC4-25		0.5		8	0.44	58.03	±7.48	5.12	±0.79	5.24	±0.01	46.45	±3.50
recC5-25		0.4		0	0	52.39	±2.57	2.48	±0.52	3.27	±0.20	46.12	±2.54
recC6-25		0.4		2	0.11	53.87	±3.64	3.45	±0.09	4.81	±0.44	43.56	±0.98
recC7-25		0.4		4	0.22	57.55	±6.58	5.78	±0.23	4.14	±0.01	49.55	±1.35
recC8-25		0.4		8	0.44	58.65	±3.06	5.54	±0.01	6.47	±0.07	44.79	±4.20
recC9-25	CEM II 42.5 R/B-M (S-V)	0.5		0	0	50.87	±3.06	2.70	±0.18	3.04	±0.01	41.59	±2.81
recC10-25		0.5		2	0.11	52.59	±2.00	3.28	±0.41	3.16	±0.03	47.07	±2.65
recC11-25		0.5		4	0.22	54.81	±6.25	5.19	±1.09	4.97	±0.67	43.65	±1.14
recC12-25		0.5		8	0.44	59.09	±6.42	7.87	±1.47	6.81	±0.01	40.08	±0.56
recC13-25		0.4		0	0	49.91	±2.05	2.52	±0.06	3.27	±0.11	43.99	±1.51
recC14-25		0.4		2	0.11	51.11	±8.29	3.24	±0.89	4.47	±0.57	43.47	±1.23
recC15-25		0.4		4	0.22	54.97	±8.87	5.24	±0.79	5.09	±0.43	45.60	±3.12
recC16-25		0.4		8	0.44	55.81	±3.68	6.25	±0.83	6.30	±0.48	43.04	±1.36
recC1-38	CEM I 42.5	0.5	38	0	0	50.13	±0.80	2.91	±0.87	2.68	±0.76	50.16	±3.29
recC2-38		0.5		2	0.11	51.08	±9.21	3.63	±0.07	4.62	±0.41	48.39	±1.39
recC3-38		0.5		4	0.22	54.24	±4.12	4.71	±0.12	4.64	±0.18	41.87	±0.51
recC4-38		0.5		8	0.44	55.91	±1.75	5.21	±0.60	6.78	±0.82	48.02	±1.65
recC5-38		0.4		0	0	50.35	±2.30	2.12	±0.79	3.11	±1.46	49.27	±3.22
recC6-38		0.4		2	0.11	54.93	±2.57	4.48	±0.52	4.39	±0.11	47.82	±1.54
recC7-38		0.4		4	0.22	57.49	±6.49	4.45	±0.09	4.52	±0.33	43.20	±2.32
recC8-38		0.4		8	0.44	58.12	±2.35	5.78	±0.23	5.62	±0.76	41.81	±3.45
recC9-38	CEM II 42.5 R/B-M (S-V)	0.5		0	0	50.92	±2.37	2.54	±0.01	2.57	±0.61	42.38	±3.14
recC10-38		0.5		2	0.11	52.45	±0.00	3.70	±0.18	3.57	±0.37	44.60	±1.57
recC11-38		0.5		4	0.22	58.13	±6.60	4.28	±0.41	4.57	±0.88	45.75	±1.65
recC12-38		0.5		8	0.44	59.37	±2.77	5.19	±1.09	5.96	±0.08	51.78	±0.98
recC13-38		0.4		0	0	51.82	±4.31	3.87	±1.47	2.47	±0.45	41.51	±6.05
recC14-38		0.4		2	0.11	53.18	±1.79	3.52	±0.06	5.60	±0.66	43.34	±1.07
recC15-38		0.4		4	0.22	55.69	±6.60	5.24	±0.89	5.60	±0.10	40.46	±3.25
recC16-38		0.4		8	0.44	58.36	±3.62	6.01	±1.72	6.87	±0.26	44.09	±4.11
recC1-50	CEM I 42.5	0.5	50	0	0	50.32	±1.06	2.30	±0.57	2.83	±0.76	42.64	±4.23
recC2-50		0.5		2	0.11	51.52	±2.44	3.30	±0.57	4.51	±0.19	47.08	±1.65
recC3-50		0.5		4	0.22	52.14	±3.74	4.16	±0.03	6.03	±0.53	42.45	±2.96
recC4-50		0.5		8	0.44	55.58	±1.28	6.97	±0.67	6.55	±0.45	39.13	±4.54
recC5-50		0.4		0	0	54.87	±1.82	2.26	±1.31	2.39	±0.22	42.18	±2.65
recC6-50		0.4		2	0.11	56.54	±3.29	3.81	±0.01	4.73	±0.39	48.25	±0.87
recC7-50		0.4		4	0.22	58.00	±1.53	3.14	±0.11	3.37	±0.21	49.01	±1.65
recC8-50		0.4		8	0.44	58.22	±1.53	6.47	±0.57	5.00	±0.38	42.17	±1.74
recC9-50	CEM II 42.5 R/B-M (S-V)	0.5		0	0	49.09	±1.00	2.04	±0.79	3.04	±0.22	47.51	±1.82
recC10-50		0.5		2	0.11	50.77	±3.13	3.31	±0.14	3.04	±0.28	39.09	±3.29
recC11-50		0.5		4	0.22	52.99	±3.21	5.25	±0.78	4.62	±0.85	41.90	±1.53
recC12-50		0.5		8	0.44	54.17	±1.03	6.24	±0.01	6.29	±0.09	51.66	±1.53
recC13-50		0.4		0	0	50.88	±4.15	2.27	±0.20	2.95	±0.39	41.10	±1.00
recC14-50		0.4		2	0.11	52.17	±4.44	3.10	±0.44	3.63	±0.71	39.48	±3.13
recC15-50		0.4		4	0.22	53.70	±1.84	5.09	±0.01	5.45	±0.05	45.96	±3.21
recC16-50		0.4		8	0.44	59.07	±3.98	6.54	±0.12	6.02	±0.27	46.48	±1.03

**Table 4:** Compressive Strength Changes with Fiber Addition.

Fiber Type	Typical Dosage	Strength Change	Citations
Steel	1–3% by volume	+9–18%	[27,28,29]
Polypropylene	0.2–0.5% by cement	+5–15% (modest)	[29]
Basalt	0.1% by cement	+11%	[30,31,32]
Carbon	1% by volume	+10%	[33,34]
Aluminum	0.04–0.05% by vol.	+10%	[35]
Natural (NFRP)	External wrap	Significant	[36]

**Table 5:** Secant modulus of elasticity Changes with Fiber Addition.

Fiber Type	Typical Dosage	Modulus Change	Citations
Steel	0.5–2% by volume	Slight increase or neutral	[29,37]
Polypropylene	0.2–1% by cement	Decrease or neutral	[38,39,40]
Basalt/PVA Hybrid	0.25% total	Up to +35%	[40,41,42]
Natural (Abaca)	0.15% by volume	+15%	[36]
PVC	1% by cement	Increase, then decrease	[43]

**Table 6:** Flexural Strength Changes with Fiber Addition.

Fiber Type/Combination	Typical Dosage	Flexural Strength Change	Citations
Steel	1–3% by volume	+50–180%	[29,37]
Polypropylene (PP)	0.15–0.2% by cement	+10–30% (modest)	[38,39,40]
Hybrid (macro + micro)	Varies	+127–443%	[44,45]
Fiber alignment (longitud.)	N/A	Up to +104%	[36,46,47]

**Table 7:** Splitting Tensile Strength Changes with Fiber Addition.

Fiber Type/Combination	Typical Dosage	Splitting Strength Change	Citations
Steel	0.5–2% by volume	+41–162%	[27,28]
Polyester	0.2–0.3% by volume	+41–66%	[9,12,29,48]
Polypropylene	0.5% by volume	+43%	[40,41]
Basalt/Hybrid	Varies	+22–29% (hybrid)	[12,42,49,50]
Natural (Coconut, etc.)	1–1.5% by mass	Significant, optimal at 1.5%	[36,51,52]

**Table 8:** Comparative Table: Residual Flexural Strength Enhancement.

Fiber Type	Typical Dosage	Flexural Strength Increase	Toughness/Residual Strength	Citations
Synthetic (PP)	2.5% by volume	+26.9%	4.75× toughness	[54,58]
Basalt	0.1–0.5% by volume	+20.8–43.5%	4.64× toughness	[9,30,42,53]
Glass	2.5% by volume	+27.9%	4.86× toughness	[55,56]
Carbon	Not specified	Highest among all	Superior residual strength	[3,12,57,59,60]

**Table 9:** Principal strain maps obtained from DIC technique for concretes incorporating recovered carbon fibers, evaluated across varying mixture parameters and loading levels of 0.1 *P_max_*, 0.5 *P_max_*, 0.9 *P_max_*, and *P_max_*.

*Series*	0.1 *P_max_*	0.5 *P_max_*	0.9 *P_max_*	*P_max_*	
*recC1-25*	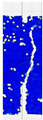	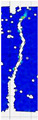	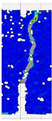	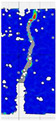	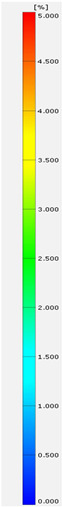
*recC2-25*	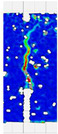	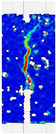	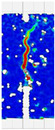	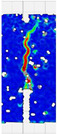	
*recC3-25*	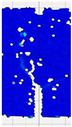	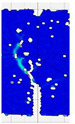	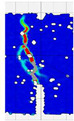	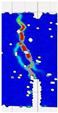	
*recC4-25*	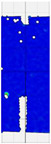	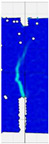	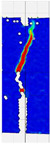	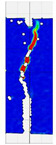	
*recC5-25*	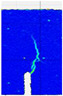	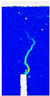	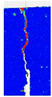	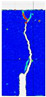	
*recC6-25*	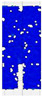	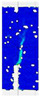	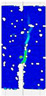	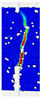	
*recC7-25*	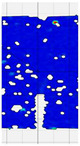	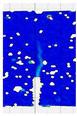	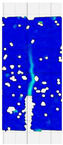	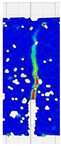	
*recC8-25*	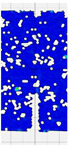	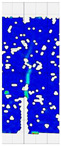	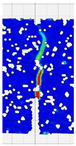	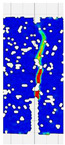	
*recC9-25*	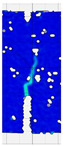	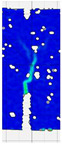	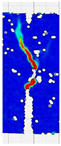	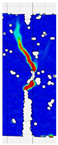	
*recC10-25*	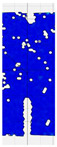	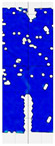	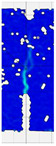	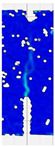	
*recC11-25*	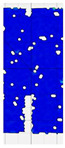	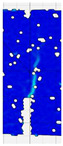	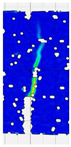	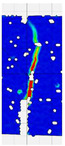	
*recC12-25*	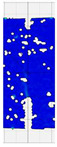	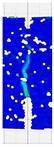	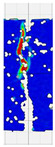	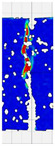	
*recC13-25*	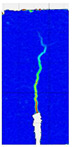	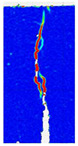	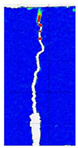	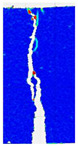	
*recC14-25*	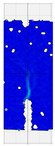	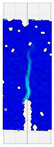	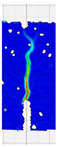	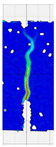	
*recC15-25*	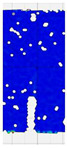	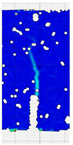	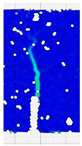	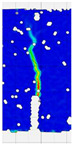	
*recC16-25*	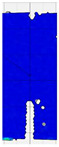	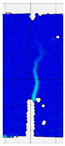	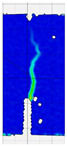	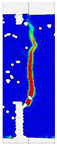	
*recC1-38*	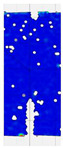	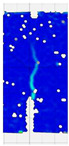	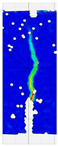	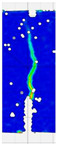	
*recC2-38*	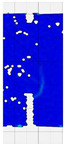	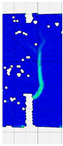	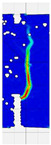	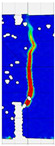	
*recC3-38*	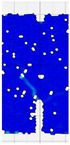	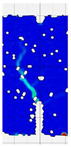	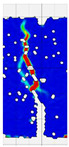	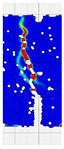	
*recC4-38*	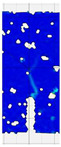	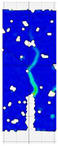	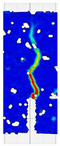	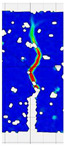	
*recC5-38*	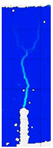	*-*	*-*	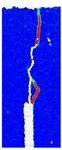	
*recC6-38*	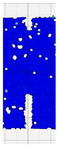	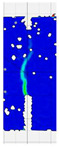	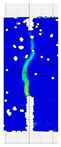	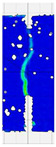	
*recC7-38*	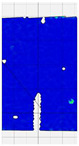	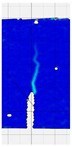	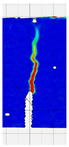	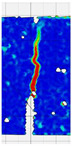	
*recC8-38*	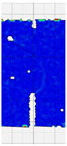	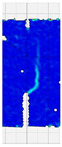	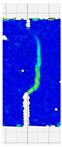	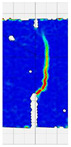	
*recC9-38*	*-*	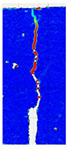	*-*	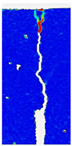	
*recC10-38*	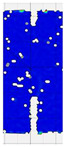	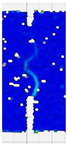	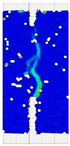	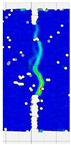	
*recC11-38*	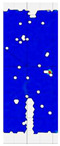	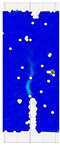	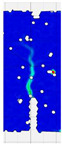	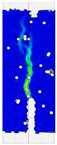	
*recC12-38*	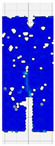	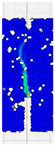	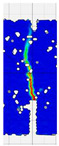	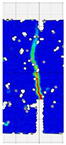	
*recC13-38*	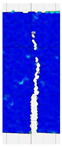	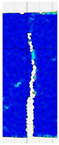	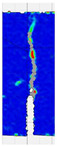	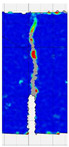	
*recC14-38*	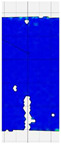	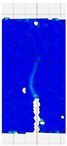	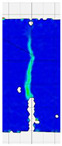	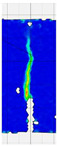	
*recC15-38*	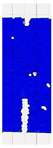	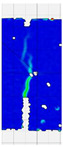	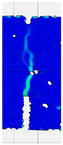	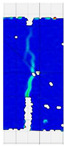	
*recC16-38*	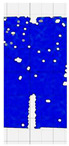	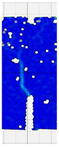	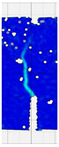	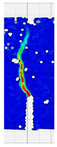	
*recC1-50*	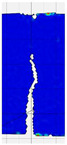	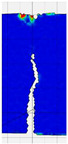	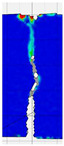	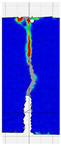	
*recC2-50*	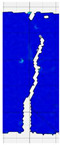	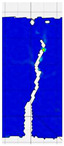	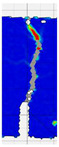	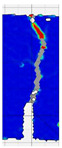	
*recC3-50*	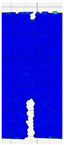	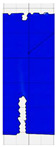	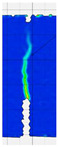	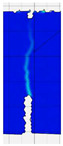	
*recC4-50*	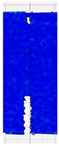	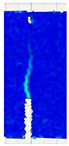	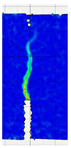	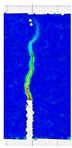	
*recC5-50*	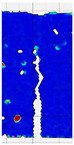	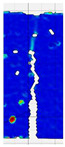	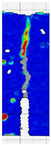	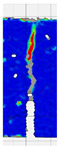	
*recC6-50*	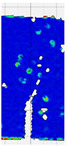	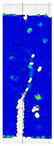	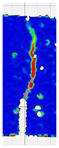	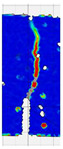	
*recC7-50*	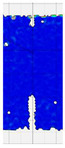	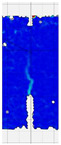	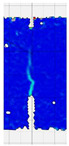	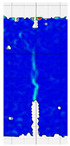	
*recC8-50*	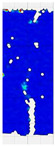	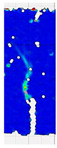	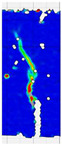	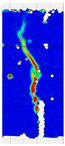	
*recC9-50*	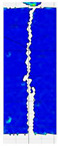	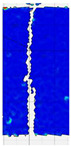	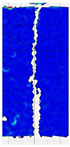	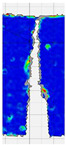	
*recC10-50*	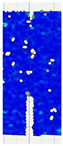	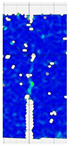	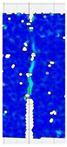	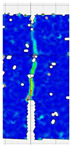	
*recC11-50*	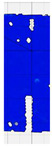	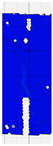	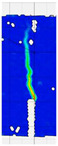	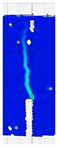	
*recC12-50*	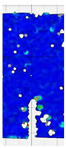	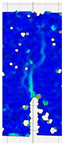	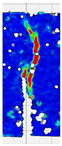	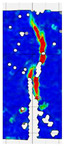	
*recC13-50*	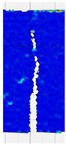	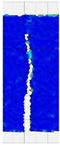	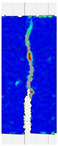	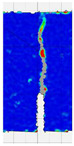	
*recC14-50*	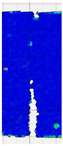	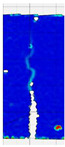	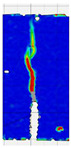	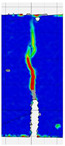	
*recC15-50*	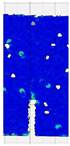	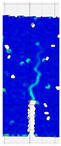	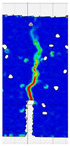	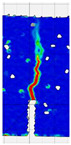	
*recC16-50*	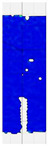	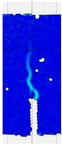	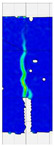	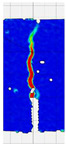	

## Data Availability

The original contributions presented in this study are included in the article. Further inquiries can be directed to the corresponding author.
